# Predictive value of cardiac electrophysiological balance index for recurrent atrial fibrillation after ablation

**DOI:** 10.1590/1806-9282.20240875

**Published:** 2024-12-02

**Authors:** Weifeng Yao, Gengling Shi, Yunfei Liu, Xixi Dai, Yang Wu

**Affiliations:** 1 Shanghai Baoshan District Wusong Central Hospital, Department of Cardiovascular Medicine – Shanghai, China.

**Keywords:** Cardiac electrophysiologic technique, Paroxysmal atrial fibrillation, Ablation, Recurrence

## Abstract

**OBJECTIVE:**

This study aimed to evaluate the prognostic significance of the cardiac electrophysiological balance index in predicting the recurrence of atrial fibrillation following radiofrequency ablation.

**METHODS:**

Patients with paroxysmal atrial fibrillation undergoing radiofrequency ablation were enrolled from July 2021 to March 2023 and categorized into recurrence and non-recurrence groups based on postoperative atrial fibrillation recurrence during a 6- to 12-month follow-up. Clinical and electrocardiogram data at admission were collected, and cardiac electrophysiological balance index was calculated. Multivariate logistic regression analysis identified independent factors contributing to atrial fibrillation recurrence. Receiver operating characteristic curves assessed predictive values.

**RESULTS:**

Among 127 subjects, 36 experienced postoperative recurrence (22 paroxysmal atrial fibrillation, 10 atrial flutter, and 4 atrial tachycardia). Significant differences in hypersensitive C-reactive protein levels, QT, QRS, and cardiac electrophysiological balance index were observed between recurrent and non-recurrent groups. Multivariate analysis revealed cardiac electrophysiological balance index as an independent risk factor for recurrence (OR 1.766, 95%CI 1.415–2.204, p<0.001). Receiver operating characteristic curve analysis showed cardiac electrophysiological balance index’s predictive value with an area under the curve of 0.865 (95%CI 0.807–0.923, p<0.001), and a cutoff value of 4.3 demonstrated a sensitivity of 87.67% and a specificity of 71.23%.

**CONCLUSIONS:**

The cardiac electrophysiological balance index emerges as a non-invasive tool with substantial predictive value for estimating the likelihood of paroxysmal AF recurrence post-ablation.

## INTRODUCTION

Atrial fibrillation (AF) is a heart arrhythmia characterized by irregular and rapid contractions of the atrium, leading to the accumulation of blood and formation of thrombosis, thereby increasing the risk of stroke and other cardiovascular events^1^. Global epidemiological surveys indicate that the worldwide incidence of AF is approximately 1.5–2.0%^2^. Recently, the morbidity of AF has been on the increase due to the growing aging population, posing a significant threat to patients’ life safety. Current clinical treatments for AF primarily involve ventricular rate control, anticoagulation, and cardioversion. Ventricular rate control methods mainly consist of β-receptor blockers, calcium channel blockers, digoxin, and other medications. Anticoagulant treatment involves the use of warfarin, dabigatran, or rivaroxaban based on the guidance of the CHA2DS2-VASc score proposed by the European Society of Cardiology in 2010^3^. However, patients who do not respond to antiarrhythmic drugs may require radiofrequency ablation to maintain sinus rhythm^4^. European Society of Cardiology Guidelines for the Management of AF suggested that catheter ablation (CA) could be regarded as a first-line treatment for preventing AF recurrence and improving symptoms in patients with paroxysmal AF^5^. However, clinical data demonstrate that the recurrence rate of AF patients after 1–5 years of RF ablation is approximately 11–29%, and with multiple recurrences, the risk of progressing to persistent AF increases. Therefore, identifying relevant indicators for predicting recurrence after RF ablation in AF patients is highly significant for improving patient prognosis^6^. The cardiac electrophysiological balance index (iCEB) is an index used to evaluate the balance of cardiac electrophysiology. It assesses the electrophysiological state of the heart by analyzing electrocardiographic data and has shown promise in predicting the potential risk of arrhythmia in recent years^7^. However, there is currently no available literature on the predictive value of EBI for recurrence after ablation of idiopathic AF. This study focuses on exploring the predictive value of EBI for recurrence after ablation of idiopathic AF, providing insights for clinical prevention and treatment.

## METHODS

### Study design and population

The study was conducted on patients who received radiofrequency ablation for paroxysmal AF at our hospital between July 2021 and March 2023. The selection criteria were as follows: (1) AF onset was documented using a 12-lead body surface electrocardiogram or a 24-h ambulatory electrocardiogram prior to the operation. The duration of AF was less than 7 days, and patients who spontaneously converted to sinus rhythm or after treatment were diagnosed with paroxysmal AF. (2) Patients met the indications for radiofrequency ablation. (3) This was the first radiofrequency ablation procedure for the patients. (4) Patients who were admitted to participate in this project and had complete clinical data during follow-up were included. Exclusion criteria included the following: (1) transesophageal echocardiography confirms the presence of a thrombus in the left atrium; (2) previous history of CA or permanent pacemaker implantation; (3) severe valvular heart disease, hepatorenal insufficiency, or thyroid dysfunction; (4) bleeding tendency or allergy to anticoagulants; and (5) patients with missing data were also excluded. This study adhered to the principles outlined in the Helsinki Declaration, and all patients provided informed consent for their participation.

### Data collection

Data for this study were collected retrospectively from medical records and laboratory databases. At admission, venous blood samples were collected for analysis using an AU5800 automatic biochemical analyzer (Beckman Coulter, USA) to measure serum levels of hypersensitive C-reactive protein (hs-CRP), low-density lipoprotein cholesterol (LDL-C), high-density lipoprotein cholesterol (HDL-C), total cholesterol (TC), and triglyceride (TG). Echocardiography measurements of left ventricular ejection fraction (LVEF) and left atrial diameter (LAD) were performed prior to surgery.

### Cardiac electrophysiological balance index measurement

The 12-lead electrocardiograph was used to collect the ECG data of all patients, and a professional in the electrocardiogram room analyzed the results following a standardized protocol. As suggested in previous research^8^, lead V4 was used to measure three consecutive and full QRS and QT intervals. The QT interval is the time from the beginning of the QRS complex to the end of the T wave, reflecting the whole process of depolarization and repolarization of the ventricular myocardium, and it was corrected using the Bazett formula. The calculation formula for iCEB is QT/QRS.

### Postoperative follow-up

Following the discharge, patients diagnosed with AF were actively monitored through telephone or outpatient services. These follow-up sessions occurred at 3, 6, and 12 months post-operation, with subsequent sessions taking place every 6 months. The purpose of these sessions was to assess symptoms and conduct ECG examinations to identify instances of postoperative recurrence. Recurrence was defined as the presence of AF, atrial flutter, or atrial tachycardia lasting ≥30 s, as detected by either a 12-lead ECG or dynamic ECG^9^.

### Statistical analysis

The Statistic Package for Social Science (SPSS) 23.0 statistical software (IBM, Armonk, NY, USA) was employed to perform data analysis. For normally distributed measurement data, mean±standard deviation (SD) was used as the expression. Two independent sample t-tests were used for intergroup comparison of data. The counting data was presented as [n(%)], and a chi-square test was used to determine. Further multifactorial logistic regression analysis was used to investigate the factors affecting the recurrence of paroxysmal AF after ablation. The predictive value of iCEB in recurrence after ablation of paroxysmal AF was assessed through the construction of a receiver operating characteristic (ROC) curve using GraphPad 8.0 software. The observed difference was deemed statistically significant at a level of significance of p<0.05.

## RESULTS

### General information and demographic characteristics

A total of 127 participants were monitored for a period of 6–12 months, with an average follow-up duration of 8.15±1.67 months. Among them, 36 individuals experienced postoperative recurrence, consisting of 22 instances of paroxysmal AF, 10 cases of atrial flutter, and 4 occurrences of atrial tachycardia. The level of hs-CRP exhibited a significant disparity between the group with recurrence and the group without recurrence. However, there were no notable distinctions in terms of gender, age, disease duration, and underlying disease type between the recurrent and non-recurrent groups (p>0.05) (Table 1).

**Table 1:** Comparison of general data between recurrent group and non-recurrent group.

Index	Recurrent group (n=36)	Non-recurrent (n=91)	χ^2^/t	p
Gender				
Male	25 (69.44%)	61 (67.03%)	0.069	0.793
Female	11 (30.56%)	30 (32.97%)		
Age (years)				
<60	23 (63.89%)	64 (70.33%)	0.496	0.481
≥60	13 (36.11%)	27 (29.67%)		
Basic diseases				
Hypertension	14 (38.89%)	36 (39.56%)	0.005	0.944
Diabetes	9 (25.00%)	20 (21.98%)	0.134	0.715
Coronary artery disease	21 (58.33%)	49 (53.85%)	0.210	0.647
Course of disease (years)	6.67±2.03	5.80±1.62	2.533	0.179
hs-CRP (mg/L)	9.78±1.76	8.64±1.98	3.014	0.003
LDL-C (mmol/L)	2.59±0.82	2.77±0.70	1.243	0.216
HDL-C (mmol/L)	1.26±0.41	1.16±0.36	1.356	0.178
TC (mmol/L)	4.06±0.84	4.32±0.82	1.599	0.122
TG (mmol/L)	2.28±0.39	1.93±0.70	2.827	0.051
LVEF (%)	57.00±8.21	62.86±12.83	2.539	0.181
LAD (mm)	38.27±2.37	38.11±2.81	0.302	0.763

hs-CRP: hypersensitive C-reactive protein; LDL-C: low-density lipoprotein cholesterol; HDL-C: high-density lipoprotein cholesterol; TC: total cholesterol; TG: triglyceride; LVEF: left ventricular ejection fraction; LAD: left atrial diameter.

### Comparison of cardiac electrophysiological balance index between recurrent group and non-recurrent group

There was a noteworthy increase in the levels of QT, QRS, and iCEB in the recurrent group compared to the non-recurrent group (p<0.05). The results indicated that in the non-recurrent group, the ECG changes that occur could be used as a predictor (Table 2).

**Table 2:** Comparison of cardiac electrophysiological balance index between the recurrent and non-recurrent groups (x̅±s).

Index	Recurrent group (n=36)	Non-recurrent (n=91)	t	p
QT (ms)	407.56±28.93	382.84±22.28	5.161	<0.001
QRS (ms)	102.82±10.69	86.09±9.21	8.808	<0.001
iCEB	4.87±0.72	4.21±1.35	2.777	0.006

iCEB: cardiac electrophysiological balance index.

### Multivariate logistic regression analysis of recurrence after ablation of paroxysmal AF

By including hs-CRP and iCEB as separate variables and considering recurrence after ablation of paroxysmal AF as the outcome (assigned as 0 for no recurrence after ablation and 1 for postoperative recurrence), the results of multivariate logistic regression analysis indicated that iCEB (OR 1.766, 95%CI 1.415–2.204) independently contributed as a risk factor for recurrence after ablation of paroxysmal AF (p<0.001).

### Receiver operating characteristic curve analysis of cardiac electrophysiological balance index predicting recurrence of paroxysmal AF after ablation

**Figure 1:**
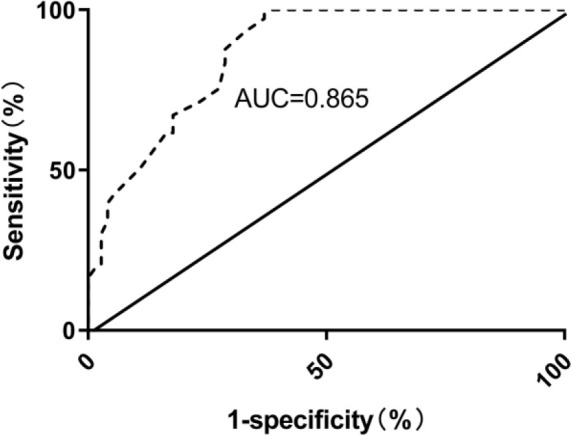
Receiver operating characteristic curve of cardiac electrophysiological balance index predicting recurrence after ablation of paroxysmal atrial fibrillation.

The findings from the ROC curve analysis revealed that iCEB had a substantial predictive value (area under the curve (AUC)=0.865, 95%CI 0.807–0.923) in determining the recurrence of paroxysmal AF post-ablation. The optimal threshold for iCEB was identified as 4.3, with a sensitivity of 87.67% and a specificity of 71.23%, as indicated in Figure 1.

## DISCUSSION

AF, characterized by its abrupt onset, dangerous condition, and rapid progression, is the most prevalent critical illness in cardiovascular medicine^10^. Radiofrequency ablation, an interventional procedure commonly used to treat AF, employs focused radiofrequency energy to cauterize abnormal heart tissue responsible for the irregular electrical signal transmission pathways leading to AF. This treatment approach boasts a success rate ranging from 50 to 80%^11^. However, it is worth noting that postoperative pulmonary vein reconnection can occur in certain patients, contributing to recurrence. In our study, the follow-up period revealed a total of 36 recurrence cases, aligning with previous reports in existing literature^12^. Research has previously emphasized that recurrent paroxysmal AF after ablation can manifest as various symptoms, including arrhythmia, palpitations, dyspnea, chest tightness, and fatigue, and may even lead to severe complications such as heart failure and stroke^13^. As a result, patients may require additional procedures or alternative treatments to effectively manage AF, leading to an increased burden, both medically and psychologically. Thus, the early prediction of recurrence after ablation for paroxysmal AF holds great significance in improving patient prognosis.

Cardiac wavelength (λ) refers to the distance that depolarized waves travel during the functional refractory period. It is determined by the product of conduction velocity (CV) and effective refractory period (ERP). Previous studies have emphasized the role of cardiac wavelength in the occurrence of arrhythmia^14^. In cases of AF, the cardiac wavelength is typically short, resulting in rapid depolarization and repolarization of cardiac muscle cells and unstable transmission of electrical signals. This promotes the persistent attack of AF. A goal of AF ablation is to lengthen the cardiac wavelength by destroying the abnormal conduction path of electrical signals. This restores the normal depolarization and repolarization times of cardiac muscle cells to restore a normal rhythm. The evaluation of cardiac wavelength is useful for predicting the recurrence of paroxysmal AF after ablation, but it requires invasive detection and is not easily applied on a large scale in clinical practice^15^. In recent years, clinicians have started using iCEB as an alternative to measuring cardiac wavelength. iCEB consists of two components, namely, QT and QRS. The QT ­interval on an electrocardiogram (ECG) is often used to assess the risk of drug-induced arrhythmia. The QRS duration reflects ­ventricular activation time on the ECG and provides good repeatability with a variation rate of less than 5%. Excessive changes in iCEB can cause significant cardiac electrophysiological imbalance, leading to arrhythmia^16^. A cohort study demonstrated a close correlation between QT and ERP and concluded that iCEB=QT/QRS is a more effective predictor of the risk of drug-induced arrhythmias compared to QT alone or dispersion of transmural repolarization^17^. This ratio serves as a simple and effective non-invasive substitute for cardiac wavelength assessment. Adali et al.^18^ found that a lower iCEB is associated with a higher ventricular extrasystole, indicating that iCEB can be used as a new non-invasive marker to predict ventricular extrasystole load in patients with normal cardiac structure. Therefore, as a non-invasive and easily measurable parameter reflecting cardiac wavelength, iCEB can play a significant role in predicting arrhythmic diseases like AF and reflecting changes in cardiac electrophysiology.

The findings indicated notable discrepancies in the levels of hs-CRP, QT, QRS, and iCEB between the two groups (p<0.001). Furthermore, multivariate logistic regression analysis revealed that iCEB is an independent risk factor for the recurrence of paroxysmal AF after ablation, indicating a strong correlation between iCEB and the recurrence of paroxysmal AF post-ablation. Furthermore, ROC curve analysis revealed that iCEB exhibited an AUC of 0.865 in predicting recurrence following ablation of paroxysmal AF. At an iCEB value of 4.3, the sensitivity was 87.67% and the specificity was 71.23%. These findings suggest the potential utility of iCEB as a valuable predictor of recurrence following ablation of paroxysmal AF.

## CONCLUSION

iCEB represents a novel non-invasive marker with considerable potential in predicting the recurrence following ­paroxysmal AF ablation. However, this study does have certain limitations, notably the limited sample size and relatively short-term follow-up duration. Going forward, efforts will be made to address these ­limitations by expanding the sample size, extending the follow-up period, and incorporating additional laboratory indicators. These measures aim to further validate the practicality of iCEB.
